# On Materials the Best Adapted for Filling the Teeth

**Published:** 1852-07

**Authors:** Wm. Robertson


### ARTICLE VIII.

*On Materials the best adapted for Filling the Teeth.* By WM. ROBERTSON, ESQ.

THE existing cause of what has been termed decay of the teeth, has long since ceased to be a disputed question.

In July, 1835, when the first edition of my work was published, and which has now arrived at a fifth edition, the subject of controversy was, whether the *cause* of the destruction of the teeth be attributable to inflammatory action, or to chemical agency. This is no longer a disputed question. It is now admitted by every practical and intelligent member of our profession, that the destruction of the teeth is occasioned by chemical agency, and *not* by inflammatory action. It is now clearly perceived that the attack upon the teeth is always made from without, and never from within, and it is equally clear, that the mischief never begins upon the smooth and level parts of the tooth, but is always found to commence in pits, fissures and interstices upon their surfaces—receptacles where the more liquid portions of the food are admitted and retained until decomposition takes place. The deposit having undergone this change, acquires a property and power, similar to an acid, of acting upon the phosphate of lime and animal matter, the two substances of which the teeth are composed. The result of this action is the destruction of the power of cohesion which previously existed between these particles, consequently the structure becomes changed from a state of crystallization into its component parts.

That this is the real cause of the destruction of the teeth, is clearly evinced from the very nature of the operations which have so long been proved effectual in remedying the evil. For instance, in using the file, our object is to remove the cavity or resting place where the food was retained, and by making a plain and smooth surface, no future lodgment can take place, and the mischief is arrested.

In filling the teeth, the object and principle is the same as in filing them: for the destructive matter is removed from its resting place, and permanently excluded by filling up the cavity firmly and securely with a durable substance that is not liable to corrode or injure the structure of the tooth, and the evil is remedied. The consideration then is, what substances are the best adapted for this purpose?

Pure and well prepared gold leaf is undoubtedly the best and the most durable material which has yet been discovered for filling the teeth, and particularly so when the operation is performed in the early stages of decay, and before the slightest tenderness has been felt in the tooth. I should, therefore, recommend gold to be used in all cases where it is practicable, in preference to any other filling. But we have numerous cases which present themselves where gold cannot be used, and where an amalgam becomes a necessary substitute.

It too often happens that filling is neglected until decay has made considerable progress. The cavity having become larger, the surrounding walls thin, and the tooth so tender, as not to admit of the pressure necessary to condense the gold into a secure and solid plug without the risk of producing pain or splitting the tooth. There are also various cases arising from the position of the decayed parts, and the shallowness, or saucer shaped form of the cavity, where a gold filling would not remain, and where it becomes necessary to use an amalgam.

The consideration then is, what materials are the best to form this amalgam?

I have given much time and labor to the investigation of this subject, and after numerous experiments with the various metals, have come to the conclusion, that gold, silver, platina and tin, in their pure state, are the best; because, I have found, in using them, either separately or combined, that they resist the acids of the mouth better than any of the other metals. An amalgam of these, however, cannot be formed without mercury; but the smaller the portion of it the better, so that there is just sufficient to unite and hold in permanent combination, the purer metals above specified.

The amalgams in general use, even those of the better class, which are composed of the metals above mentioned, contain a great deal too much mercury; and I shall presently show that the cause for this superabundance of mercury, arises from the incorrect proportioning of the metals and the improper mode of mixing them.

The method generally adopted in preparing an amalgam, is to combine the mercury at once with the other metals, so that the compound may be in a state of readiness for using; and when a portion is required, it has to undergo a process of heating and rubbing to bring it back from its crystallized form to a pasty state before putting it into the tooth.

Instead of adopting this method, the mercury ought to be kept separate from the other metals, and only mixed with them at the time of using. This mode of mixing with the metals, rightly proportioned, will produce a harder and more durable filling, and the metals will combine with one-third of the mercury required in the former mode of preparing the amalgam.

As an illustration of this fact, even in the better class of amalgams, I may refer to a paper that appeared in the Pharmaceutical Journal for March, 1850, on amalgams for stopping the teeth, by Arnold Rogers, Esq.

"I have much pleasure," he says, "in communicating through the Pharmaceutical Society, a few remarks on amalgams, as well as the formula for the one I have used for some years, and although occasionally some specimens have come under my observation which seemed to possess qualities superior to the general characrer of amalgams; yet, on the whole, I have been satisfied with my own compound, not but that I think it may be improved upon; and, when leisure time will allow me, I shall endeavor to profit by the hints of my professional brethren."

After a few remarks on various amalgams, Mr. Rogers proceeds to point out the method of preparing his own. And first, an amalgam is composed of silver one part, and mercury four parts. This amalgam of silver is to be united with an amalgam of gold, which consists of gold one part, mercury three parts. These are to be kept in separate boxes, and when used, the proportions are to be two parts by weight of the gold amalgam to one of the silver; thus making the amalgams, when combined, to consist of silver one part, gold two parts, and *mercury ten parts.* Then, as to the manner of using it. "The two pellets had better be triturated thoroughly in a mortar before the compound is submitted to the flame of a spirit lamp; and I have observed," he says, "that for the better incorporation, it is necessary to submit it to the heating and trituration, in the usual way, three or four times. The cavity of the tooth being quite prepared for the reception of the amalgam, all the mercury that can be pressed out between the thumb and fingers should be so separated, and the compound immediately packed in the cavity."

Now I feel persuaded, that Mr. Rogers will agree with me as to the desirability of getting rid of a portion of this mercury; and I shall presently show that by using the same metals, with the addition of another, and by proportioning them and preparing the amalgam differently, we shall be enabled to dispense with *seven parts of this mercury out of the ten.*

Some dentists use an amalgam of palladium and mercury, which makes a firm and durable filling; but it becomes black in the tooth.

A few years ago, cadmium was introduced as a filling, and for a short time it promised well, and appeared to be an improvement upon former amalgams; but after a few months trial, it showed its defects and was very properly discarded. It neither retained its color, nor its hardness, for when acted upon by the friction of the opposing teeth, it soon wore away, and when not acted upon, it became, in parts, yellow as saffron; with the disadvantage, also, of having a strong tendency to produce galvanic action in the mouth. Objectionable as this compound is, it still continues to be used, by a certain class of practitioners, under the title of "Parisian Filling."

There are various kinds of amalgams used by the same class of individuals, which contain copper, lead, bismuth, zinc, and antimony, all of which are bad.

To test an amalgam properly, is a work of time; a few months is not sufficient, years of observation are required to ascertain its real character and to judge of its utility. Its introduction to the public, ought, therefore, to be the act of an experienced member of the profession; for it becomes rather a serious matter, if we find that the filling we may have been using pretty freely for six or twelve months, upon the strength of another's recommendation, should turn out, after the manner of the cadmium preparation, a complete failure.

The amalgam which I am about to describe, contains nothing novel, so far as the materials are concerned. There are no new metals introduced; they have all been used in different forms for many years; but it is in the proportioning of them, and by a different method of preparing the compound, that I have been enabled of late years to get rid of two parts out of three of the mercury formerly required; and this I consider to be an important advantage; for as I have before said, an amalgam cannot be formed without mercury, but the smaller the portion required of it the better.

Since making this change, I have had ample time, upwards of three years, to test the merits of the compound, and can, therefore, speak practically of its utility.

The amalgam consists of gold, one part; silver, three parts; and tin, two parts; and it is of the utmost importance that the metals should be pure—free from the smallest particle of alloy.

The mode of uniting the metals is, first to put the gold and silver into the crucible, and just as they are at the point of melting, add the tin, which requires less heat than the former.

When they become melted, but not over heated, pour them from the crucible; melt them a second time for the purpose of having them properly mixed, and then pour them into an ingot, of a shape the best adapted for reducing the compound into the finest powder.

The mercury is to be kept apart from the powder, and mixed with it at the moment the amalgam is to be put into the tooth.

The mercury must be perfectly pure and the quantity required will be equal in weight to the powder; and here we get rid of *seven parts of the mercury out of the ten before mentioned.*

In mixing the powder with the mercury, the exact proportions of each can be ascertained to the greatest nicety, and by a very simple process, which will save time and material, and which, I believe, makes a firmer compound, than by using a larger portion of mercury and squeezing part of it out again.

A measure for the mercury may be made from a square portion of ivory, by drilling in it three holes, one may be made to contain four grains, the second eight, and the third, twelve grains. The mercury to be poured into either of these as more or less may be required to fill the cavity of the tooth, and by drawing the finger across so as to bring the mercury on a level with the surface of the ivory, the portion wanted is accurately obtained.

The measure for the powder is equally simple and convenient. It is a metal tube, with a handle attached, calculated to hold four grains, which is also got to a nicety by filling and then drawing the finger across the mouth of the tube, so as to make level measure.

The mercury should be kept in a small bottle with an India rubber tube attached to the neck of it, and the mercury is readily pressed through the tube into the measure. In this way the exact quantity of each of the metals is readily obtained

The powder and mercury to be emptied from the measure into a small glass mortar, and rubbed till they become mixed. The compound is then to be rubbed firmly with the finger in the hollow of the hand, till converted into a paste, which, before hardening, allows plenty of time for inserting it into the tooth.

I have found this compound to be far superior to the former class of amalgams. It does not, like palladium, and some of the other metals that have been used, become black in the mouth. It retains its hardness and firmness of texture, and is not like the cadmium, liable to be worn away by the friction of the teeth.

Formerly, in fixing artificial teeth upon a gold plate, it was necessary to avoid bringing the gold in contact with a tooth filled with an amalgam; because a portion of the mercury entered into the gold and destroyed it, this evil is now remedied by the compound containing so small a quantity of mercury, which is held in permanent combination with the other metals, so that not a particle of *it* is abstracted by the embrace of a gold clasp. With these important advantages over former amalgams, it is also used with much greater facility.

From experiments which I have made during the last twelve months, I find that the quantity of mercury which I have named, can be reduced still further, and which, I believe, will be an improvement upon the present compound; but as I have before stated, more time and practical experience, to test the result of even this alteration, is desirable before taking the responsibility of giving it to the public.

THE SQUARE, BIRMINGHAM,

*May 1st*, 1852.

